# Supplementary material for: Effective deep learning training for single-image super-resolution in endomicroscopy exploiting video-registration-based reconstruction
Source: Int J Comput Assist Radiol Surg. 2018 Apr 23;13(6):917–24. doi: 10.1007/s11548-018-1764-0 (PMC5973979; doi:10.1007/s11548-018-1764-0)
Supplement: Supplementary file 1 — Supplementary material 1 (pdf 22 KB) [file 11548_2018_1764_MOESM1_ESM.pdf]

The movie called “SR_pCLE.avi” shows the sample results presented in the “Effective deep learning training for single-image super-resolution in endomicroscopy exploiting video-registration-based reconstruction” submitted at IPCAI 2018.
The movie shows pCLE sequence in 5 different configurations. All sequences were built from the validation set used for evaluating the performance of SR models we trained for pCLE super-resolution.
The sequences represent as follows (from left to right):
1. low-resolution input images from the validation-set
2. super-resolved output of the EDSR -based model
3. super-resolved output of the FSRCNN-based model
4. super-resolved output of the SRGAN-based model
5. high-resolution estimation of the input images generated by the mosaicking technique
Sequences are presented in two rows: the first row shows full image, the second row shows zoom for the central part of the image.

The link to the movie below (web viewer):
https://drive.google.com/file/d/1FgoLLiStiA0mQpQsJ0wLK2CfFcy_EdlM/view
